# Faunistic updates on Greek ants: First record of *Strumigenys membranifera* (Emery, 1869) from Crete and *Strumigenys baudueri* (Emery, 1875) from Andros

**DOI:** 10.3897/BDJ.14.e183362

**Published:** 2026-05-12

**Authors:** Antonios Loulakis, Christos Georgiadis, Dimitrios Kollaros, Jakovos Demetriou, Ioannis Chasourakis, Ioannis Zografakis, Emmanouil Kabourakis

**Affiliations:** 1 Olive, Vine & Agroecological Production Systems Lab Hellenic Mediterranean University (HMU), Heraklion, Greece Olive, Vine & Agroecological Production Systems Lab Hellenic Mediterranean University (HMU) Heraklion Greece; 2 Section of Zoology-Marine Biology, National and Kapodistrian University of Athens, Zografou, Greece Section of Zoology-Marine Biology, National and Kapodistrian University of Athens Zografou Greece; 3 Zoology Museum of the University of Athens, Zografou, Greece Zoology Museum of the University of Athens Zografou Greece; 4 National and Kapodistrian University of Athens, Athens, Greece National and Kapodistrian University of Athens Athens Greece; 5 Joint Services Health Unit Cyprus, BFC RAF Akrotiri, Limassol, Cyprus Joint Services Health Unit Cyprus, BFC RAF Akrotiri Limassol Cyprus; 6 Enalia Physis Environmental Research Centre, Acropoleos 2, Aglantzia 2101, Nicosia, Cyprus Enalia Physis Environmental Research Centre, Acropoleos 2, Aglantzia 2101 Nicosia Cyprus

**Keywords:** *
Strumigenys
*, olive orchards, Crete, tramp species, Myrmicinae

## Abstract

**Background:**

The genus *Strumigenys* Smith, 1860 (Formicidae, Myrmicinae) comprises over 890 species worldwide. *Strumigenys membranifera*, a widely distributed tramp species, is known for its small size, thelytokous parthenogenesis and predatory behaviour.

**New information:**

Herein, we report the first occurrence of *S. membranifera* from the island of Crete, Greece. Specimens were collected in olive orchard agroecosystems, located in the Messara Plain, using pitfall traps during surveys conducted in 2023 and 2024. In addition, we report the first occurrence of *S. baudueri* from the island of Andros, Greece, where specimens were collected from leaf litter. These records expand our knowledge of *Strumigenys* species on the island of Crete and the Aegean Islands and contribute to our understanding of the ecology and distribution of native and non-native members of this genus in Greece.

## Introduction

The ant genus *Strumigenys* Smith, F., 1860 (Formicidae, Myrmicinae) includes more than 890 species ( Bolton 2025) and is the third most diverse ant genus, characterised by its small size, cryptobiotic habits and specialised predatory behaviour ( Hamer et al. 2021). Most species forage in soil, leaf litter or rotten wood, while some species are arboreal. Additionally, many species are specialised predators with trap-jaw mandibles that primarily hunt Collembola and other small soft–bodied arthropods (Masuko 1984, Gronenberg 1996). Distinctive morphological characters, that make the genus easily recognisable include mandibular adaptations, spongiform tissue on the metasoma, a dorso-ventrally flattened head, a diverse range of specialised pilosity and a reduction in antennomere segments, which occurs in both short and long mandibular forms ( Hamer et al. 2021).

The genus *Strumigenys* is predominantly distributed across tropical and subtropical zones. Within Europe, four species are considered native: *S. argiola* Emery, 1869, *S. baudueri* Emery, 1875, *S. tenuipilis* Emery, 1915 and *S. tenuissima* Brown, 1953 ( Janicki et al. 2016, Seifert 2018, Hamer and Turner 2024). In addition to these, six non-native species have also been recorded: : *S. perplexa* Smith, 1876, *S. emmae* Emery, 1890, *S. lewisi* Cameron, 1886, *S. membranifera* Emery, 1869, *S. rogeri* Emery, 1890 and *S. silvestrii* Emery, 1906 ( Wetterer 2011, Wetterer 2012, Borowiec 2014, , Hamer et al. 2021, , Michlewicz 2022, Hamer and Turner 2024). *S. rogeri* has so far been reported only from indoor environments, whereas *S. membranifera* and *S. silvestrii* have established populations outdoors, predominantly in the warmer southern, Mediterranean regions of Europe (, Wetterer 2011, , Hamer and Turner 2024). Amongst European *Strumigenys*, all four native species are known in Greece; however, amongst the non-native species, only *S. membranifera* has been detected in the Epirus Region ( Demetriou et al. 2023, Borowiec and Salata 2025).

*Strumigenys membranifera* is a small, cryptic myrmicine ant with a wide distribution across subtropical and tropical regions worldwide. Workers are small (1.9–2.1 mm) with triangular mandibles; they also have tiny teeth on pliers–like mandibles that can clamp down and hold prey ( Wetterer 2011). Known as a tramp species, it often colonises disturbed habitats and is frequently associated with human activity. They reproduce asexually through thelytokous parthenogenesis ( Ito et al. 2010).

In Crete, we report the first record of *S. membranifera*, found in olive orchard agroecosystems. In other countries, *S. membranifera* has primarily been reported from open, sun-exposed habitats with moist soils and plentiful leaf litter, including scrublands, areas beneath isolated shrubs and pasturelands. The species exhibits strong synanthropic tendencies and is most frequently found in disturbed or human-modified environments ( Borowiec and Salata 2025).

## Materials and methods

**Figure 1.**
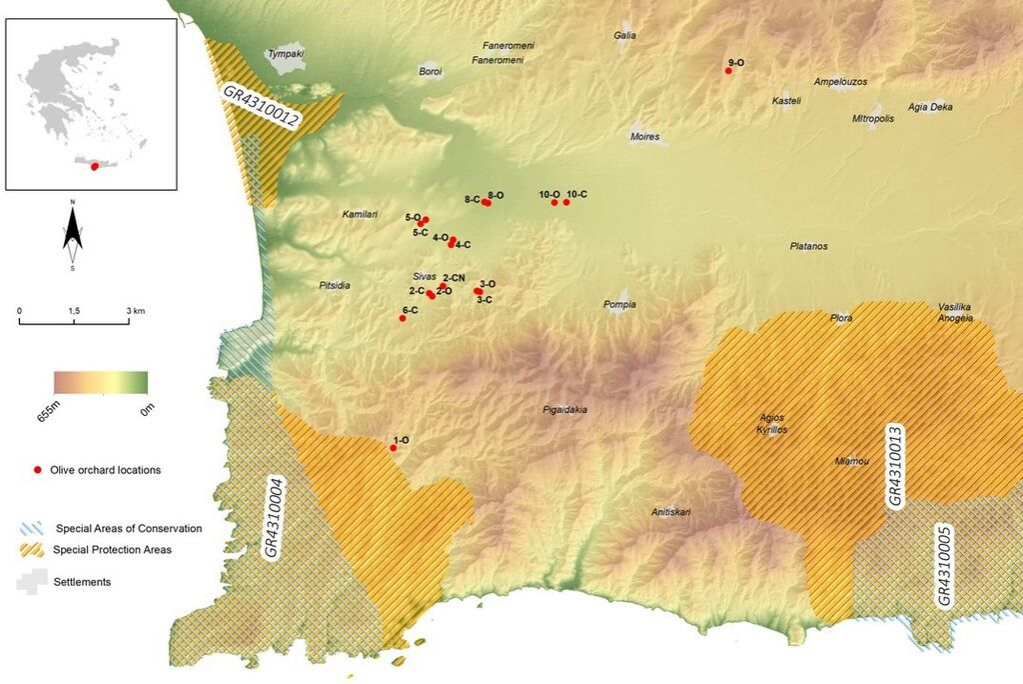
Map of the survey area in Crete showing olive orchards (8-O, 9-O, 2-C and 10-C) where *S. membranifera* specimens were collected.

The specimens of *S. membranifera* were found during a myrmecological survey conducted in olive orchards in the western part of Messara Plain, Crete, Greece. The survey included 16 olive orchards, located in both hilly and plain agroecological zones (Fig. 1). The specimens were collected using pitfall traps containing propylene glycol, during 2023 and 2024. In each olive orchard, five traps (10 traps/ha) were placed under the olive tree canopy. Specimen identification was performed under a stereomicroscope (Olympus DSX1000 Digital Microscope), based on morphological characteristics following Borowiec and Salata (2025). The collected specimens are deposited in the Laboratory of Olive, Vine and Agroecological Production Systems of the Hellenic Mediterranean University in Heraklion, Crete and the Zoology Museum of the University of Athens, Greece.

The specimens of *S. baudueri* were found in a survey conducted by Georgios Kakiopoulos, MD, in leaf litter during November 2016 in Andros Island. Two specimens were recovered by placing leaf litter into an overnight sifter over a plastic box. The ants were slow-moving and, when disturbed, held close to the edge of the box. Specimens were photographed using a Zeiss stereomicroscope mounted with a ProgRes C3 3.2-megapixel CCD sensor digital camera and stack/montage photos were produced using the Helicon Focus 8 software (depth map rendering method). The collected specimens are deposited in the Zoology Museum of the University of Athens, Greece.

## Taxon treatments

### Strumigenys membranifera

Emery, 1869

1FA4D5F4-33EF-5AB4-B41C-47D03BB93B38

#### Materials

- **Type status:** Other material. **Occurrence:** recordedBy: Loulakis Antonios; individualCount: 2; sex: female; lifeStage: worker; occurrenceID: 80A0D92B-DA7D-503C-8F2B-543867D070CF; **Taxon:** scientificName: *Strumigenys membranifera*; genus: Strumigenys; specificEpithet: membranifera; scientificNameAuthorship: Emery, 1869; **Location:** country: Greece; stateProvince: Crete; locality: Messara Plain, Heraklion Prefecture in Crete; verbatimElevation: 51 m; verbatimCoordinates: 35°02'09.2"N 24°49'40.1"E; decimalLatitude: 35.0359; decimalLongitude: 24.8278; **Event:** samplingProtocol: pitfall traps; eventDate: 7/2/2023; **Record Level:** institutionCode: HMU LOVAPS
- **Type status:** Other material. **Occurrence:** recordedBy: Loulakis Antonios; individualCount: 1; sex: female; lifeStage: alate queen; occurrenceID: 920ED149-C1B9-5E47-89AA-7AC576447F8C; **Taxon:** scientificName: *Strumigenys membranifera*; genus: Strumigenys; specificEpithet: membranifera; scientificNameAuthorship: Emery, 1869; **Location:** country: Greece; stateProvince: Crete; locality: Messara Plain, Heraklion Prefecture in Crete; verbatimElevation: 258 m; verbatimCoordinates: 35°04'05.5"N 24°54'04.0"E; decimalLatitude: 35.0682; decimalLongitude: 24.9011; **Event:** samplingProtocol: pitfall traps; eventDate: 9/2/2023; **Record Level:** institutionCode: HMU LOVAPS
- **Type status:** Other material. **Occurrence:** recordedBy: Loulakis Antonios; individualCount: 1; sex: female; lifeStage: alate queen; occurrenceID: D43D3BAA-EABE-574F-BAB3-32FF1B1A515A; **Taxon:** scientificName: *Strumigenys membranifera*; genus: Strumigenys; specificEpithet: membranifera; scientificNameAuthorship: Emery, 1869; **Location:** country: Greece; stateProvince: Crete; locality: Messara Plain, Heraklion Prefecture in Crete; verbatimElevation: 99 m; verbatimCoordinates: 35°00'49.7"N 24°48'37.1"E; decimalLatitude: 35.0138; decimalLongitude: 24.8103; **Event:** samplingProtocol: pitfall traps; eventDate: 7/1/2024; **Record Level:** institutionCode: HMU LOVAPS
- **Type status:** Other material. **Occurrence:** recordedBy: Loulakis Antonios; individualCount: 1; sex: female; lifeStage: alate queen; occurrenceID: 1EF8A644-DA5B-53CA-87D9-8FBF0635240B; **Taxon:** scientificName: *Strumigenys membranifera*; genus: Strumigenys; specificEpithet: membranifera; scientificNameAuthorship: Emery, 1869; **Location:** country: Greece; stateProvince: Crete; locality: Messara Plain, Heraklion Prefecture in Crete; verbatimElevation: 51 m; verbatimCoordinates: 35°02'10.3"N 24°51'05.4"E; decimalLatitude: 35.0362; decimalLongitude: 24.8515; **Event:** samplingProtocol: pitfall traps; eventDate: 7/1/2024; **Record Level:** institutionCode: HMU LOVAPS

#### Distribution

Introduced globally to all continents, except Antarctica, *S. membranifera* appears to disperse easily to new areas through human-mediated activities. In Greece, the species was previously known only from Epirus ( Salata and Borowiec 2018, Demetriou et al. 2023). However, *S. membranifera* has a broad distribution across Europe and the Mediterranean Basin, occurring in southern Europe, North Africa and the eastern Mediterranean and is considered cosmopolitan ( Borowiec and Salata 2025).

#### Notes

**Figure 2.**
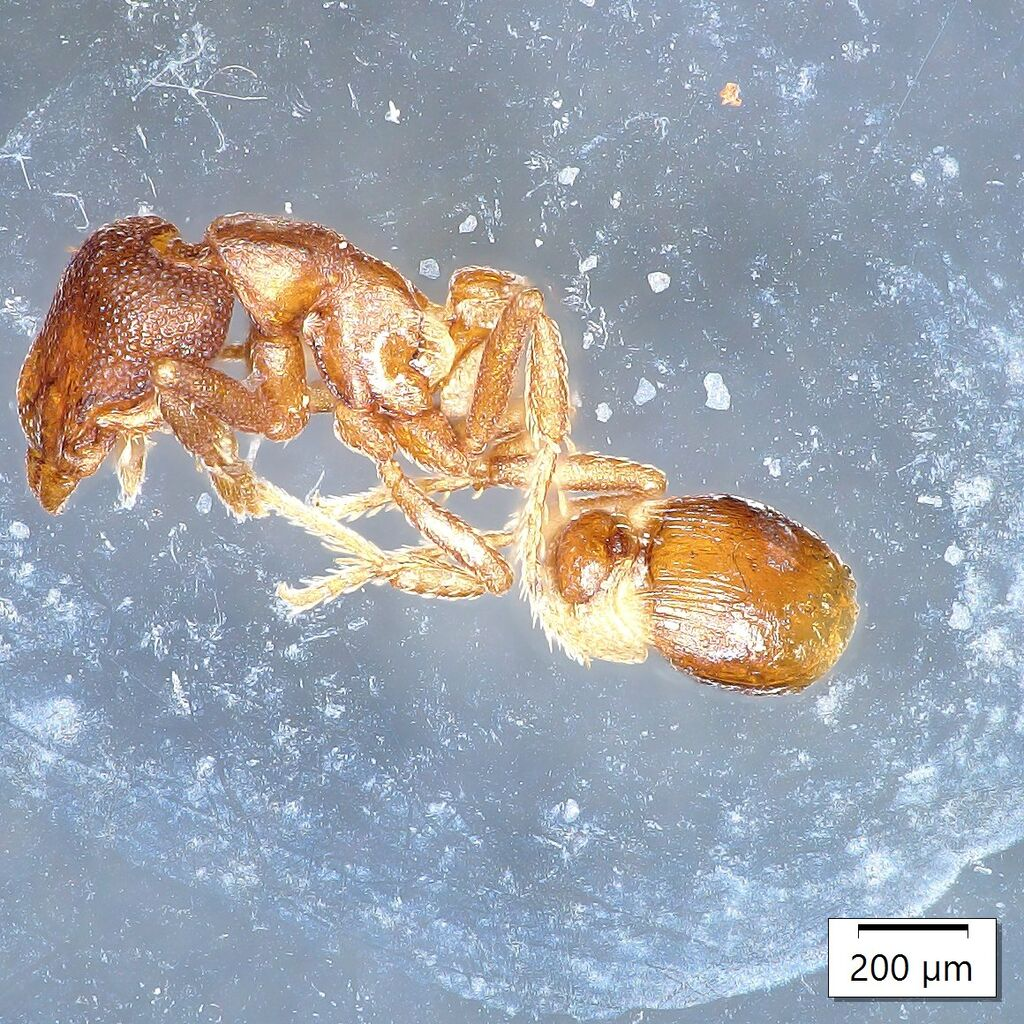
*Strumigenys membranifera* worker (source A. Loulakis, LOVAPS lab – HMU).

**Figure 3.**
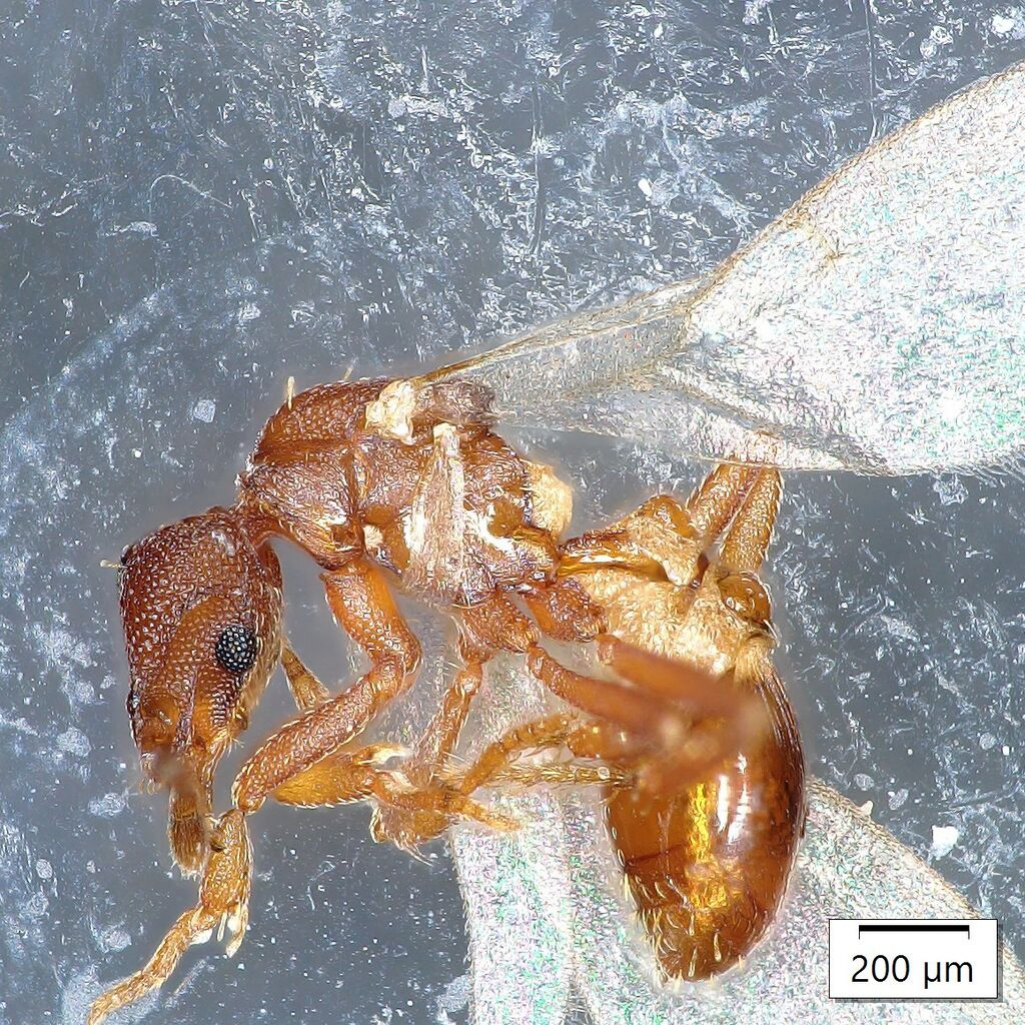
*Strumigenys membranifera* queen (source A. Loulakis, LOVAPS lab – HMU).

During a myrmecological survey of ant diversity conducted in 2023 and 2024 in olive orchard agroecosystems with applied green infrastructure (GI), located in the Messara Plain, Crete, *Strumigenys membranifera* was recorded for the first time:

- Two worker specimens in an olive orchard (8-O) near the locality of Petrokefali in July 2023 (Fig. 2);
- Additionally, one female was collected in an olive orchard (9-O) near the locality of Roufas in September 2023;
- In 2024, one female was also collected in an olive orchard near the locality of Sivas (2-C) and another in an olive orchard (10-C) near the locality of Petrokefali (Fig. 3).

*Strumigenys membranifera* was identified, based on diagnostic morphological characters described in the monographic review of the ants of Greece by Borowiec and Salata (2025). The species is characterised by its small body size, cordiform head and short triangular mandibles with a serially dentate margin (12 teeth), along with a distinctive transverse mandibular depression behind the basal tooth. Additional diagnostic features include a broad, projecting clypeus, flattened antennal scapes bearing spatulate hairs, strongly developed spongiform appendages on the waist and at the base of the first gastral sternite and the absence of long standing setae on the dorsal mesosoma and first gastral tergite.

### Strumigenys baudueri

Emery, 1875

61E70B66-AF31-5EBA-8B92-5D72E70E56CE

#### Materials

- **Type status:** Other material. **Occurrence:** recordedBy: Kakiopoulos, Georgios; individualCount: 2; sex: female; lifeStage: worker; occurrenceID: 56A5A1C8-5780-5961-BDFE-EB5E1AEB9879; **Taxon:** scientificName: *Strumigenys baudueri*; genus: Strumigenys; specificEpithet: baudueri; scientificNameAuthorship: Emery, 1875; **Location:** country: Greece; stateProvince: Central Aegean Islands; locality: Andros, Vitali; verbatimElevation: 180 m; verbatimCoordinates: 37°55'45.5"N 24°48'03.2"E; decimalLatitude: 37.9293; decimalLongitude: 24.8009; **Event:** samplingProtocol: leaf litter; eventDate: 11/14/2016

#### Distribution

*Strumigenys baudueri* has been recorded in Greece from Epirus and Sterea Ellas (Bolton 2000, as *Pyramica baudueri*). Outside Greece, the species is distributed across Europe and the Mediterranean Basin, including Algeria, Armenia, Bulgaria, Croatia, France (mainland), Hungary, Italy (mainland, Sardinia, Sicily), Malta, Morocco, Serbia, Spain (mainland), Switzerland, Tunisia and Türkiye (Borowiec and Salata 2025).

#### Notes

**Figure 4.**
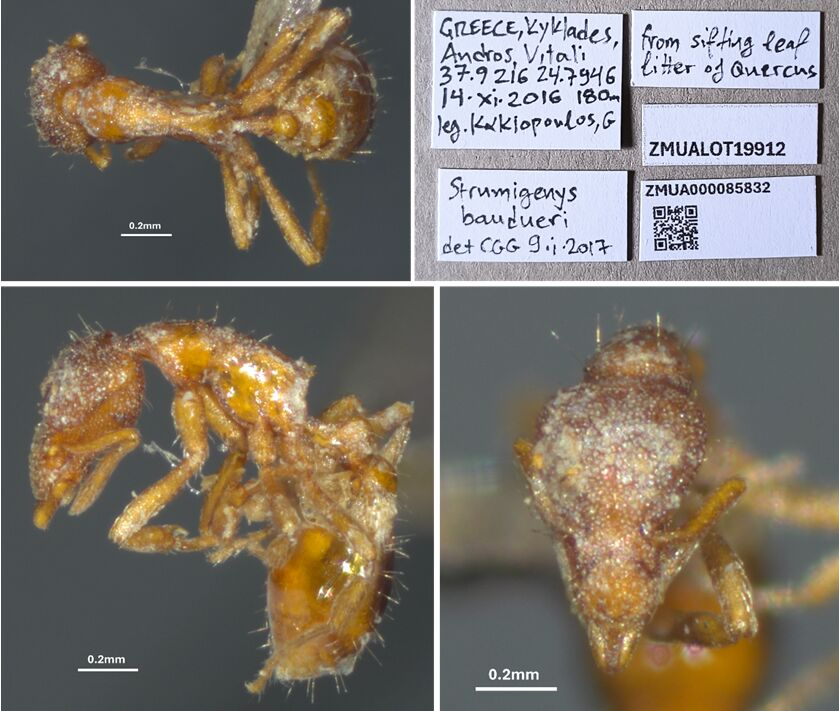
*Strumigenys baudueri* worker. Upper left: Dorsal view, lower left: Lateral view, Lower right: Head view. Upper right: specimen labels with metadata.

Additionally, two more specimen records of *S. baudueri* are reported for the first time from insular Greece (Fig. 4); specifically, from the island of Andros (Cyclades). These specimens are deposited at the Zoology Museum of the University of Athens (ZMUA).

## Discussion

This article presents the first documented record of the alien *S. membranifera* from Crete, the first report of *S. baudueri* from Andros and the first report of both species from the Aegean Archipelago as a whole. Our findings contribute to the growing knowledge of the myrmecofauna of Crete, which to date, includes 107 known ant species ( Salata et al. 2020, Borowiec and Salata 2022, Borowiec et al. 2023, Schifani et al. 2024, Demetriou et al. 2025,Borowiec and Salata 2025, García 2025). Nearly 18 ant taxa found i Borowiec and Salata 2025n Crete are endemic to the Island ( Salata et al. 2020), while 10 additional alien species have been recorded ( Demetriou et al. 2023). Further studies are required to assess the environmental impacts of this alien ant on native biodiversity.

Additionally, this study provides a new record of the native *S. baudueri* from Andros, a large Aegean island in the Cyclades, for which a comprehensive checklist is lacking ( Scupola and Borowiec 2023). According to Borowiec and Salata (2025), “no data on the biology of this species from Greece and neighbouring countries” are available. The collected specimen of *S. baudueri*, sifted from *Quercus* leaf litter, provides some insight on habitats needed to be further investigated in order to uncover further distributional data on *S. baudueri*, as well as other leaf litter ants in Greece.

The specimens of *S. membranifera*, were collected from olive orchards, characterised by a herbaceous layer dominated by *Oxalis pes-caprae* and *Capparis spinosa*, together with species of the Poaceae, Asteraceae and Fabaceae families. Olive orchard agroecosystems show pronounced seasonal variation in vegetation structure. During the wet season, in spring, the floristic community reaches its peak (approximately 35 species) and is dominated by *Oxalis pes-caprae* and species of the Poaceae family, such as *Cynodon dactylon* and *Avena* sp. During the dry period, species richness is lower (≈ 10 species) with *Capparis spinosa*, *Cynodon dactylon*, *Piptatherum miliaceum* and *Setaria verticiliata* becoming dominant in the floristic community. Specimens of *S. membranifera* were collected from multiple localities, offering insights into the distribution of this species and its habitat. This suggests that *S. membranifera* is spreading and may now be naturalising in olive orchard agroecosystems in Crete, Greece, which cover approximately 25% of the island’s total land area.

## Supplementary Material

XML Treatment for Strumigenys membranifera

XML Treatment for Strumigenys baudueri
